# Efficacy and safety of acupuncture as an adjuvant therapy for osteoporosis: a systematic review and meta-analysis of randomized controlled trials

**DOI:** 10.3389/fendo.2025.1561344

**Published:** 2025-05-09

**Authors:** Zixin Teng, Jingwei Zhu, Kuiwu Li, Tingting Tong, Wei Li, Haoran Chu, Peiyang Sun

**Affiliations:** ^1^ Second Clinical Medical College of Anhui University of Traditional Chinese Medicine, Hefei, China; ^2^ Department of Encephalopathy, Second Affiliated Hospital of Anhui University of Traditional Chinese Medicine, Hefei, China; ^3^ Mingyi Hall, Second Affiliated Hospital of Anhui University of Traditional Chinese Medicine, Hefei, China

**Keywords:** acupuncture, osteoporosis, adjuvant therapy, meta-analysis, systematic review

## Abstract

**Objective:**

To systematically evaluate the efficacy and safety of acupuncture as an adjuvant therapy for osteoporosis (OP) through a comprehensive synthesis of recent randomized controlled trial (RCT) evidence.

**Methods:**

A systematic literature search was conducted across PubMed, Web of Science, CNKI, and Wanfang databases (2014 – 2024) to identify RCTs investigating acupuncture combined with conventional therapy for OP. Study quality was appraised using the Cochrane Risk of Bias tool, and meta-analyses were performed using RevMan 5.4 and Stata 15.0, with subgroup analyses stratified by intervention type, population characteristics, and treatment duration.

**Results:**

28 RCTs (n=2,758) were included. Meta-analysis revealed acupuncture significantly enhanced bone mineral density (BMD) versus controls: total (SMD = 0.47, *p* = 0.03), femoral neck (MD = 0.05, *p* = 0.01), lumbar spine (SMD = 0.40, *p* < 0.001), Ward’s triangle (MD = 0.07, *p* = 0.02), and hip (SMD = 0.55, *p* < 0.001), with particularly marked improvements in the postmenopausal osteoporosis subgroup. Acupuncture demonstrated significant improvements in treatment efficacy, biochemical markers, pain scores, and symptom assessments, while reducing adverse events. Warm needle moxibustion outperformed controls in femoral neck (MD = 0.07, *p* = 0.002) and hip BMD (SMD = 0.87, *p* < 0.001), while electroacupuncture significantly elevated serum calcium (MD = 0.18, *p* = 0.02). Short-term interventions (≤ 3 months) demonstrated optimal efficacy.

**Conclusion:**

Acupuncture demonstrates efficacy and safety as an OP adjuvant therapy. Current evidence is limited by regional bias and methodological heterogeneity. Multicenter, large-sample RCTs are needed to standardize protocols and validate long-term therapeutic efficacy.

**Systematic review registration:**

https://www.crd.york.ac.uk/PROSPERO/, identifier CRD42024499354.

## 1 Introduction

Osteoporosis (OP) is a bone metabolic disorder characterized by osteopenia and degeneration of bone microstructure (1). Clinical manifestations mainly involve bone pain, spinal deformity, and fragility fracture (2). Based on the etiology, OP can be categorized into primary osteoporosis (POP), which encompasses senile osteoporosis (SOP), postmenopausal osteoporosis (PMOP), and idiopathic osteoporosis (IO), as well as secondary osteoporosis (SO) (3). As the global population is steadily aging, the prevalence of OP is witnessing a continuous upward trend (4). According to estimates by the World Health Organization, the number of affected individuals is projected to reach 221 million by the year 2050 (5), thereby posing a significant public health burden (6).

Osteoblasts (OB) and osteoclasts (OC) work together to maintain bone metabolism balance. OCs are involved in skeletal remodeling through bone matrix resorption (7), while OBs participate in bone synthesis and mineralization, playing a role in bone formation and reconstruction (8). An imbalance favoring OC resorption over OB formation can lead to persistent bone loss and reduced bone strength (9), ultimately contributing to OP. Modern pharmacological treatments for OP include bone resorption inhibitors like bisphosphonates and calcitonin, bone formation promoters like teriparatide and romosozumab, and estrogen receptor modulators (10). Nevertheless, it should be noted that these pharmaceutical agents have been linked to a number of deleterious effects, including gastrointestinal reactions, nephrotoxicity, and an elevated risk of cancer (11). Therefore, finding a safe and effective treatment modality is crucial.

The utilization of external therapies in the realm of Traditional Chinese Medicine (TCM) has a long and significant history when it comes to the management of OP. Such external therapies encompass a diverse range, including acupuncture, moxibustion, Chinese therapeutic massage, herbal fumigation, and health-preserving exercises. Acupuncture, in particular, is recognized as a standard or adjunct therapy for OP in countries like China due to its practicality, minimal side effects, and low cost (12). Many animal studies have confirmed acupuncture’s efficacy. One study (13) demonstrated that the application of acupuncture at Zusanli (ST36), Shenshu (BL23), and Dazhui (GV14) resulted in a significant regulation of serum hormone levels in ovariectomized (OVX) rats, including estradiol, adrenocorticotropic hormone, corticotropin-releasing hormone and cortisol. This intervention inhibited weight gain in OVX rats and significantly enhanced bone mineral density (BMD) and bone microarchitecture. Another experimental study (14) found that acupuncture at bilateral BL23 in OVX rats significantly down-regulated the level of the cytokine Dickkopf-1, elevated the levels of β-catenin and Wnt3a, and influence the Wnt/β-catenin signaling pathway, thereby effectively regulating bone metabolism and slowing bone mass decline. Modern theoretical studies suggest that acupuncture modulates metabolic functions by adjusting hormone secretion and immune responses, reducing inflammation, preserving bone structure, and promoting bone formation (15).

Large-scale randomized controlled trials (RCTs) are critical to guide clinical practice and broader implementation of acupuncture for OP (16). The most recent systematic review (2018) (17) evaluating the efficacy of acupuncture for OP, while offering preliminary insights, revealed critical limitations: inadequate stratification of OP subtypes and treatment duration, insufficient safety evaluation frameworks, inadequate outcome measures and exclusion of emerging techniques, such as laser acupuncture, thick-needle therapy and acupoint catgut embedding (ACE). Therefore, updating existing evidence and providing high-quality data on the efficacy and safety of acupuncture for OP is imperative. This meta-analysis updates the evidence through rigorous synthesis of contemporary RCTs, delivering the most updated and comprehensive evidence on the efficacy-safety profile of acupuncture as an adjuvant therapy for OP.

## 2 Materials and methods

### 2.1 Registration and protocol

For the current investigation, it was meticulously prospectively documented in the International Prospective Register of Systematic Reviews, known as PROSPERO, under the registration number CRD42024499354. Moreover, the review methodology was crafted with precision, strictly adhering to the Preferred Reporting Items for Systematic Reviews and Meta-Analyses (PRISMA) guidelines. Additionally, the study also conformed to the PRISMA extension statement for meta-analysis (18), further enhancing the rigour and comprehensiveness of the research approach.

### 2.2 Search methods

**Figure 1:**
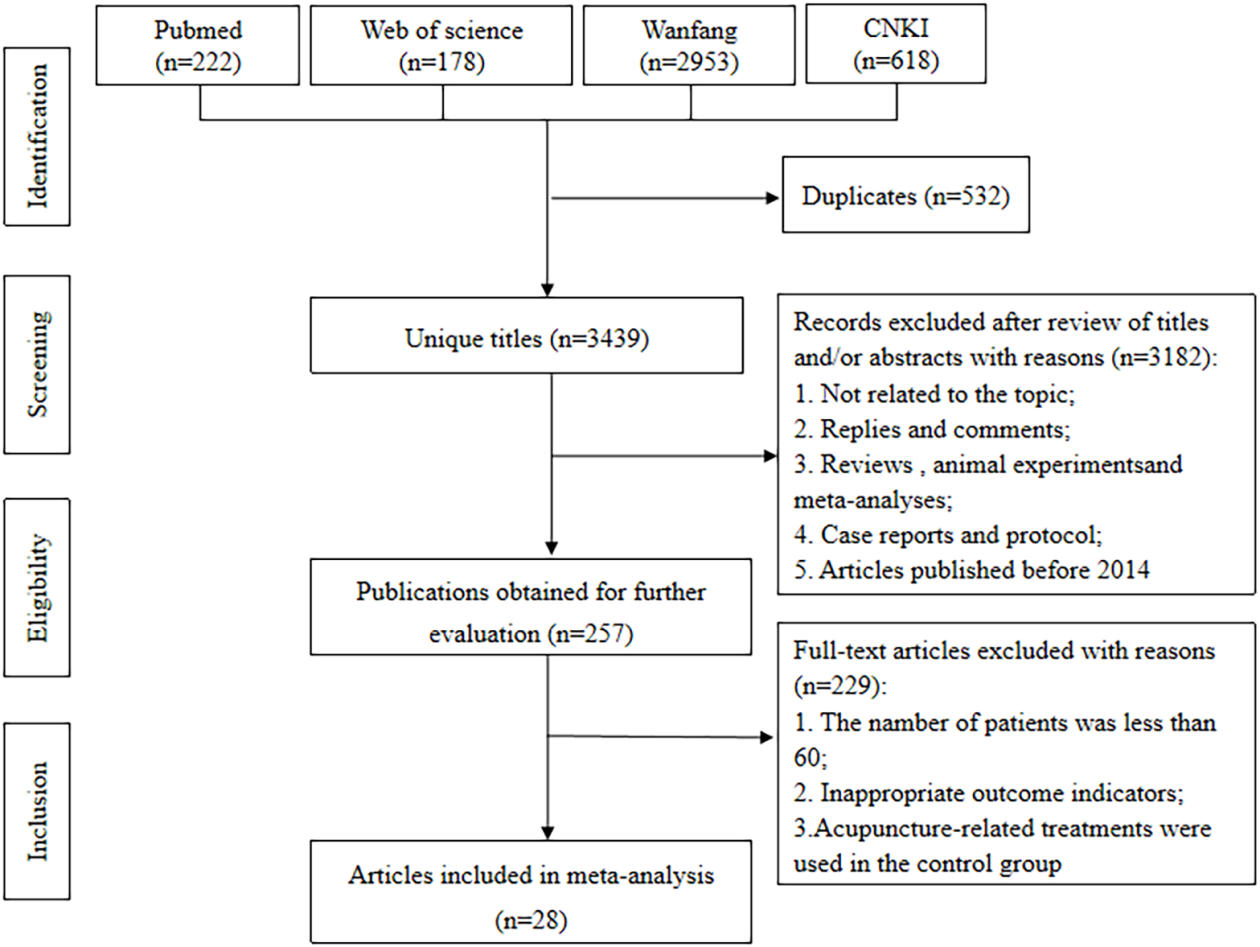
Flowchart of literature search.

A comprehensive search was conducted for RCTs investigating the adjunctive use of acupuncture for OP, covering the period from 2014 to 2024 across databases including PubMed, Web of Science, China National Knowledge Infrastructure (CNKI), and Wanfang databases. The search terms included “Acupuncture & Pharmacopuncture” and “Osteoporosis & Osteoporoses & Osteoporosis, Post-Traumatic & Osteoporosis, Post Traumatic & Post-Traumatic Osteoporoses & Post-Traumatic Osteoporosis & Osteoporosis, Senile & Osteoporoses, Senile & Senile Osteoporoses & Osteoporosis, Involutional & Senile Osteoporosis & Osteoporosis, Age-Related & Osteoporosis, Age Related & Bone Loss, Age-Related & Age-Related Bone Loss & Age-Related Bone Losses & Bone Loss, Age Related & Bone Losses, Age-Related & Age-Related Osteoporosis & Age Related Osteoporosis & Age-Related Osteoporoses & Osteoporoses, Age-Related”. No restrictions were applied with regard to either language or geography. The search strategies are illustrated in **Figure 1**.

### 2.3 Inclusion and exclusion criteria

Eligibility for the included RCTs was determined by the following criteria:

Inclusion criteria:

1. Patients with a confirmed diagnosis of POP, including PMOP, SOP, and IO), with a dual-energy X-ray absorptiometry BMD T-score ≤ -2.5.
2. Intervention group: acupuncture, manual acupuncture, scalp acupuncture, electro-acupuncture (EA), auricular acupuncture, warming needle moxibustion (WNM), body acupuncture, laser acupuncture, ACE and thick needle (either as a sole treatment or as an adjunct.). Control group: usual care, pharmacological therapies; sham acupuncture, placebo or no treatment.
EA: integrates manual needling with microcurrent waveforms replicating endogenous bioelectric signals delivered through inserted needles.
WNM: combustion of moxa cones affixed to retained needles facilitates thermal energy transfer to acupoints along the needle shaft.
ACE: implantation of biodegradable catgut sutures at acupoints using tapered needles to achieve prolonged stimulation.
Thick needle: preserves conventional filiform morphology with augmented diameters (0.4-1 mm compared to standard 0.18-0.35 mm).
Regional techniques: scalp acupuncture: precise needling of cephalic reflex zones;
hand acupuncture: microsystem therapy targeting palmar acupoints; ear acupuncture: auricular somatotopic stimulation; body acupuncture: conventional somatic point selection.
3. The primary outcome measure of BMD is reported along with secondary outcomes. These include adverse events; related biochemical indicators of bone metabolism (serum bone Gla-protein [BGP], serum alkaline phosphatase [ALP], serum calcium [GA]), serum phosphorus [P]); clinical related score table (SF-36 quality of life scale, visual analog scale [VAS], TCM syndrome score, efficacy evaluation of TCM syndromes, efficacy evaluation of OP and Oswestry Disability Index [ODI]).

Exclusion criteria:

- Review articles, animal studies, non-RCTs, retrospective studies and protocols;
- OP secondary to other diseases;
- Studies with inaccurate data or incomplete outcome measures, particularly where there is no access to the original authors for clarification;
- Studies with fewer than 60 patients enrolled;
- Publications before 2014;
- Duplicates.

### 2.4 Data extraction

Data extraction was conducted by two independent researchers, with the aforementioned criteria serving as the foundation for the process:

- Publication details (title, first author, publication year).
- Characteristics (population and follow-up duration).
- Participant information, including their number, age, gender, and the duration of OP, was gathered and analyzed.
- Intervention specifics (methods and acupoint selection).
- Outcomes, both primary and secondary, were detailed. The analysis presented continuous data as the mean and standard deviation, while categorical data were represented as event counts and the total number of participants. The criteria for evaluating the efficacy of OP treatment were as follows (1): markedly effective: significant alleviation of pain and discomfort, with an increase in BMD (2). effective: notable relief in pain and discomfort was observed, but no significant change in BMD occurred (3). ineffective: no relief or worsening of symptoms was noted, with a decrease in BMD. The total effective rate is expressed as a percentage (the sum of the markedly effective and effective cases divided by the total number of cases in the study). The TCM symptom score was usually defined using the OP grading quantitative scale, which consists of six items: low back pain, soreness and weakness of the waist and knees, pain in the lower limbs, flaccidity in the lower limbs, trudge, and dizziness. The total score was 36 points, categorized into four levels: none (2), light (2), moderate (4), and severe. The Nimodipine method of calculation involves the subtraction of the score post-treatment from the score prior to treatment, the division of the result by the score prior to treatment, then multiplying by 100%. The criteria for evaluating treatment efficacy were as follows (1): Cured: The clinical symptoms in Traditional Chinese Medicine (TCM) had either completely disappeared or been greatly alleviated, and the syndrome score was decreased by 95% or more (2). Markedly effective: significant progress has been achieved in clinical symptoms and physical signs in TCM, as evidenced by the substantial decline of at least 70% but less than 95% (3). Effective: The clinical symptoms and physical signs in TCM had shown noticeable improvement, where the syndrome score was lowered by at least 30% but less than 70% (4). Ineffective: The clinical symptoms and physical signs in TCM had not improved significantly or had even deteriorated, and the syndrome score was decreased by less than 30%. The total effective rate is expressed as a percentage (the sum of the cured, markedly effective and effective cases divided by the total number of cases in the study). Any discrepancies in the final assessments were adjudicated by a third researcher.

### 2.5 Quality assessment

The quality of the incorporated RCTs was appraised by utilizing the Cochrane risk of bias tool. Two authors independently evaluated several pivotal aspects, namely random sequence generation, allocation concealment, blinding, the completeness of outcome data, and reporting bias. Subsequently, the risk of bias was classified into one of three categories: “high,” “low,” or “unclear.” In the event of any discrepancies between the two initial authors, a third author intervened to reach a resolution.

### 2.6 Statistical analysis

The literature search was systematically conducted using EndNote X9, with data extraction and management performed in Microsoft Excel. Meta-analyses were conducted using RevMan 5.4 and Stata 15.0. Dichotomous outcomes, such as adverse events, were analyzed using risk ratios (RRs) with corresponding 95% confidence intervals (CIs). Continuous outcomes, including BMD and biochemical markers, were assessed by calculating mean differences (MDs) when measurement units were consistent across studies; standardized mean differences (SMDs) were applied for outcomes with heterogeneous units. All results were expressed with 95% CIs.

Heterogeneity was evaluated using the Cochrane Q test complemented by the *I*² statistic. A fixed-effect model was employed when the Q test indicated non-significant heterogeneity (*p* > 0.05) and *I*² ≤ 50%. In cases of substantial heterogeneity (*p* < 0.05 or *I*² > 50%), sensitivity analyses (e.g., sequential exclusion of individual studies) and subgroup analyses (e.g., stratification by osteoporosis subtype, intervention duration or intervention protocol) were performed to identify sources of variability, followed by a random-effects model for meta-analysis.

## 3 Results

### 3.1 Research and selection

Following database searches, we identified 3,971 relevant studies. After removing 532 duplicates, we screened 3,439 studies based on titles, resulting in 3,182 exclusions. Full-text reviews were performed for the remaining 257 studies, ultimately leading to the inclusion of 28 studies (19–46). As outlined in **Table 1**, basic information regarding the selected literature has been presented. **Figure 1** presents a visual representation of the selection process.

**Table 1:** Baseline characteristics of include studies.

Authors	Study period	Country	Study design	Patients (n)	Age (years)	Male (n)	Durational of disease	Median follow-up (months)
				Acupuncture/Control	Acupuncture/Control	Acupuncture/Control	Acupuncture/Control	
An (32).	2016-2017	China	prospective	37/37	62.01 ± 5.73/61.79 ± 5.82	14/15	3.15 ± 0.25/3.11 ± 0.28	1
Cai et al. (19)	2011-2013	China	prospective	43/42	51 ± 7/50 ± 6	–	–	12
Chai et al. (29)	2014–2015	China	prospective	33/32	50.26 ± 5.91/49.85 ± 5.69	22/21	14.33 ± 1.72/14.25 ± 1.67 (months)	3
Chen et al. (20)	–	China	prospective	42/44	64.35 ± 9.12/63.65 ± 8.42	14/16	5.95 ± 3.32/5.95 ± 3.32	6
Chen et al. (39)	2018-2019	China	prospective	55/55	69.3 ± 4.7/72.6 ± 5.4	27/25	4.6 ± 0.7/4.1 ± 0.6	–
Chen et al. (40)	2020-2020	China	prospective	31/32	64 ± 5/64 ± 5	–	4.1 ± 0.7/4.7 ± 1.1	3
Chen et al. (37)	2016-2018	China	prospective	48/48	63 ± 8/64 ± 8	21/18	7.21 ± 1.62/6.95 ± 1.58	3
Deng et al. (33)	2014-2016	China	prospective	50/50	63.72 ± 3.28/64.43 ± 3.64	20/22	5.16 ± 2.83/5.29 ± 2.74	1.5
Han et al. (34)	2017-2018	China	prospective	45/44	71.21 ± 5.20/71.24 ± 5.23	20/20	3.49 ± 1.15/3.42 ± 1.11	1
Huang (21).	2011-2014	China	prospective	50/50	67.8 ± 5.2/68.1 ± 4.9	14/15	1.2 ± 0.3/1.4 ± 0.2	3
Huang et al. (25)	2014-2015	China	prospective	40/40	68.52 ± 4.15/68.51 ± 4.12	22/21	–	3
Li et al. (35)	2017-2017	China	prospective	50/50	–	–	–	3
Li (26).	2012-2014	China	prospective	50/50	72.06 ± 5.71/72.37 ± 4.62	23/26	2.90 ± 0.50/2.80 ± 0.60	3
Liu et al. (22)	2011-2013	China	prospective	80/80	–	40/34	5.3 ± 1.8605/4.3 ± 1.5504	2
Liu et al. (27)	2013-2014	China	prospective	62/62	55.86 ± 6.92/56.15 ± 6.77	–	17.88 ± 2.25/18.69 ± 2.33 (months)	6
Luo et al. (28)	2015-2015	China	prospective	36/36	63.61 ± 3.43/64.03 ± 3.30	10/8	3.06 ± 1.01/2.67 ± 0.99	3
Luo (30).	2014-2017	China	prospective	45/45	67.03 ± 2.72/67.82 ± 3.02	17/18	2.05 ± 0.38/2.18 ± 0.42	3
Tian et al. (38)	–	China	prospective	32/33	65 ± 5/64 ± 6	5/4	4.40 ± 1.62/4.00 ± 1.31	3
Tian et al. (42)	–	China	prospective	36/36	66 ± 4/65 ± 5	3/5	3.50 ± 1.00/2.90 ± 1.10	6
Wang et al. (31)	2016-2017	China	prospective	91/91	62.24 ± 5.78/63.36 ± 7.58	47/42	2.25 ± 0.59/2.25 ± 0.59	3
Ye et al. (43)	2020-2022	China	prospective	40/40	68 ± 5/68 ± 5	15/16	4.76 ± 0.71/4.64 ± 0.75	2
Yuan et al. (23)	–	China	prospective	40/40	63.4 ± 4.3/61.2 ± 5.0	13/11	5.04 ± 0.36/4.98 ± 0.19	1
Zhang et al. (36)	2014-2016	China	prospective	40/40	63.39 ± 4.16/62.23 ± 4.57	17/15	1.4 ± 0.1/1.2 ± 0.2	6
Zhou et al. (24)	2012-2013	China	prospective	80/80	59.5 ± 1.31/60.1 ± 1.27	25/26	2.6 ± 0.85/2.7 ± 0.84	6
Hassan et al. (44)	2021-2022	Egypt	prospective	34/34	56.59 ± 2.03/56.53 ± 2.25	–	–	3
Huang et al. (45)	2021-2022	China	prospective	32/33	59.81 ± 2.15/59.58 ± 2.65	–	11.83 ± 3.74/10.97 ± 3.68	3
Li et al. (41)	2020-2022	China	prospective	43/43	57.06 ± 6.57/58.03 ± 6.03	–	6.98 ± 2.63/7.29 ± 2.35	3
Chen et al. (46)	2014-2017	China	prospective	116/110	59.11 ± 5.90/58.13 ± 6.02	–	–	6

### 3.2 Study characteristics

A total of 2,758 patients were encompassed by the 28 studies integrated into this analysis. Of these, 1,382 and 1,376 patients assigned to the acupuncture and control groups, respectively, with each group possessing sample sizes exceeding the threshold of 30. In the acupuncture group, the mean age of participants fluctuated between 50.26 and 72.06 years, whereas in the control group, the mean age of participants fluctuated from 49.85 to 72.60 years. Regarding the mean disease duration, it extended from 1.19 to 11.83 years in the acupuncture group and from 1.19 to 10.97 years in the control group. Treatment durations varied from 1 to 12 months. Interventions for the control group included conventional anti-osteoporosis treatments such as alendronate, Caltrate D3 tablets, alfacalcidol capsules, compound ossotide injections, salmon calcitonin, and TCM decoctions. The patients in the acupuncture group received acupuncture alone or in addition to standard treatment. Various acupuncture techniques utilized included simple acupuncture, EA, WNM, ACE, laser acupuncture, and thick needles.

The statistical analyses conducted on the acupuncture point selection within the 28 articles uncovered a total of 49 acupoints distributed across 10 meridians. The Bladder meridian of foot-taiyang (BL) emerged as one of the most frequently utilized meridians, followed by the Gallbladder meridian of foot-shaoyang (GB), Governing Vessel (GV), Conception Vessel (CV), and so on. The acupuncture points most frequently applied were: BL23, BL20, ST36, GB39, GV4.

### 3.3 Bias risk assessment results

**Figure 2:**
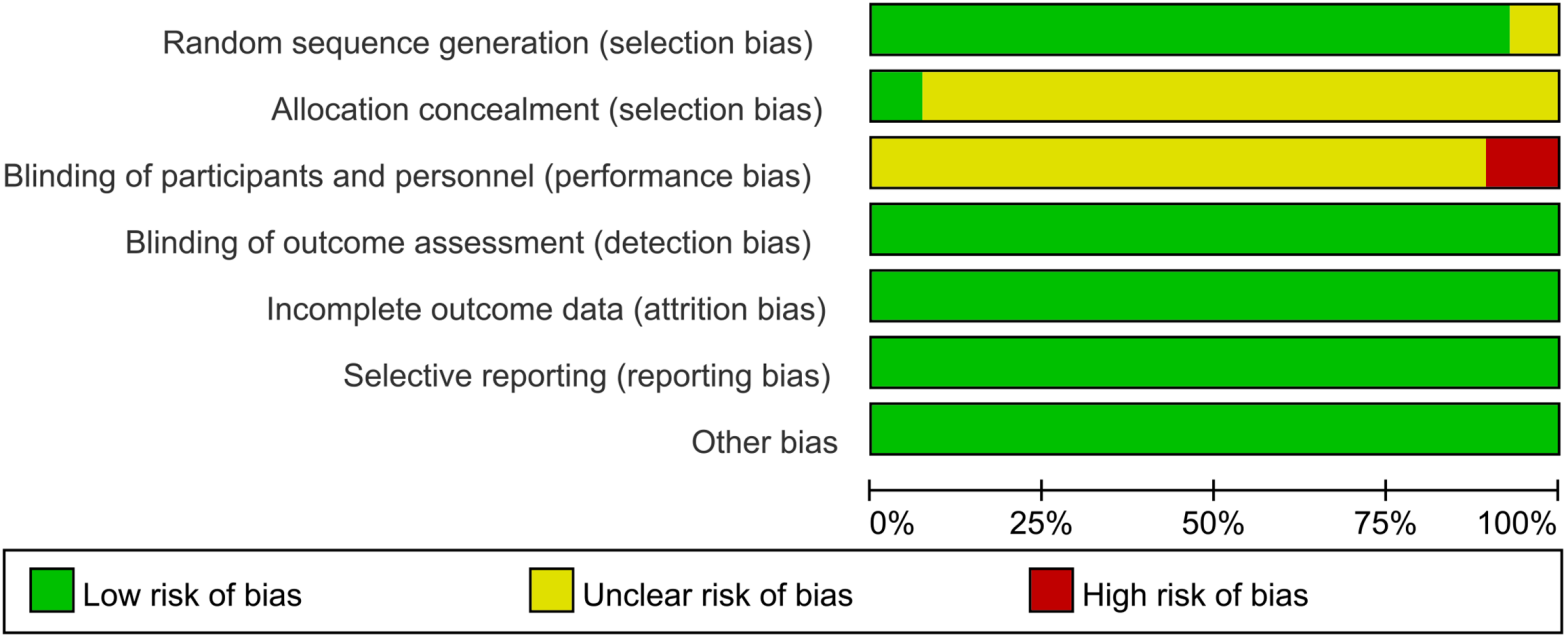
Summary of potential bias risk of included studies.

**Figure 3:**
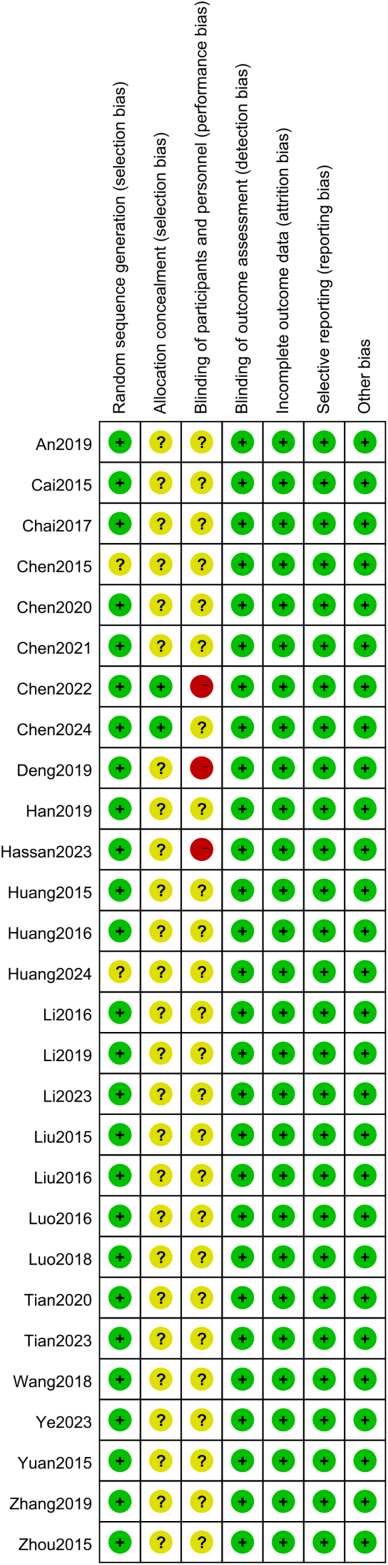
Potential risk of bias of each included study.

An evaluation of the quality of the included literature was conducted by means of the Cochrane risk assessment tool. In the 28 studies reviewed, all were reported as RCTs. The randomization method was described in 26 studies, and this was assessed as low risk. These two studies (20, 45) did not make reference to the specific allocation methods, and were consequently evaluated as being of unknown risk. These two studies (40, 46) utilized the envelope method for the purpose of allocation concealment, a process which was subsequently found to be low risk. The residual studies failed to touch upon the aspect of allocation concealment and were evaluated as having an unknown risk. As stated in these studies (33, 40, 44) single-blinding was employed, and the resulting studies were consequently categorized as high risk. The residual investigations did not make any reference to blinding procedures, which led to their being assigned an indeterminate risk rating. All the articles had complete outcome indicator data, and no other biases were found, so they led to their being assigned a low-risk rating. All the outcome indicators expected by the studies were reported and were assessed as low risk. As illustrated in **Figure 2** and **Figure 3**, the risk assessment is displayed.

### 3.4 Primary outcomes

#### 3.4.1 Change in total BMD

**Figure 4:**
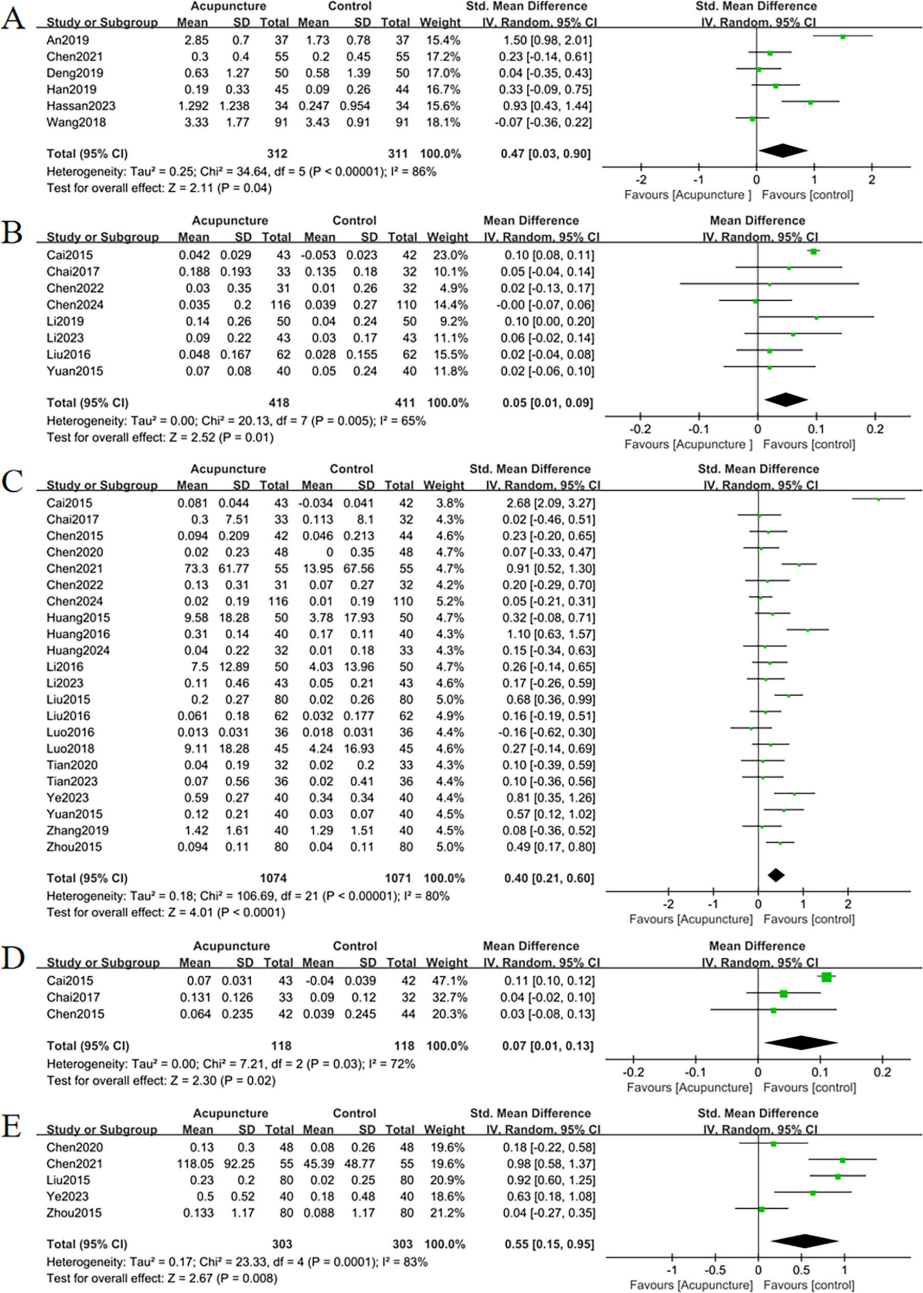
Forest plots of the main outcome measures: **(A)** change in total BMD, **(B)** change in BMD of femoral neck, **(C)** change in BMD of the lumbar spine, **(D)** change in BMD of the Waed’s triangle, **(E)** change in BMD of femur. Forest plots demonstrated that all outcomes were significantly more effective than controls (*p* <0.05).

**Figure 5:**
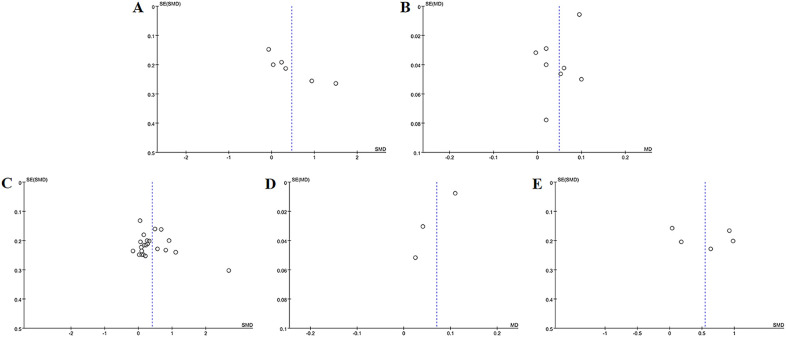
Funnel plots of the main outcome measures: **(A)** change in total BMD, **(B)** change in BMD of femoral neck, **(C)** change in BMD of the lumbar spine, **(D)** change in BMD of the Waed’s triangle, **(E)** change in BMD of hip. Funnel plots indicated publication bias for changes in total BMD and femoral neck BMD. Mild publication bias was observed for changes in lumbar spine BMD, while changes in Ward’s triangle BMD and hip BMD exhibited no publication bias.

**Figure 6:**
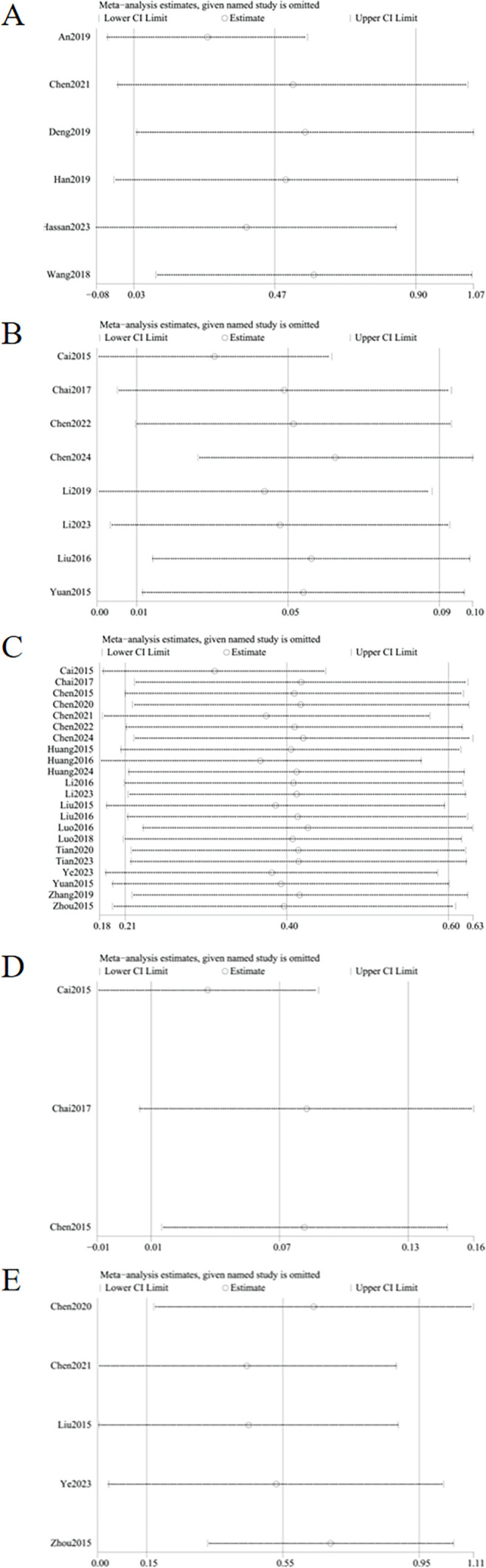
Sensitivity analysis of **(A)** change in total BMD, **(B)** change in BMD of femoral neck, **(C)** change in BMD of the lumbar spine, **(D)** change in BMD of the Waed’s triangle, **(E)** change in BMD of hip. Sensitivity analysis revealed instability in outcomes of changes in total BMD and changes in Ward’s triangle BMD. In contrast, outcomes of changes in femoral neck BMD, lumbar spine BMD, and hip BMD demonstrated stability.

The meta-analysis evaluating total BMD alterations encompassed six RCTs. Acupuncture exhibited a statistically significant advantage over the control group in enhancing total BMD (SMD = 0.47, 95% CI = 0.03 to 0.89, *I* ^2^ = 85%, *p* = 0.03) (**Figure 4**), though substantial heterogeneity was noted (*I* ^2^ = 85%). Visual inspection of the funnel plot (**Figure 5**) and Egger’s test (*p* = 0.015) suggested potential publication bias. Sensitivity analysis demonstrated that exclusion of studies 32,39,34 and 44 reduced the statistical significance to nonsignificant levels (**Figure 6**).

Subgroup analyses stratified by population characteristics revealed significant total BMD improvements in PMOP patients compared with controls (SMD = 0.93, 95% CI = 0.43 to 1.4, *I* ^2^ = NA, *p <*0.001). Conversely, no significant differences were observed in elderly-onset or primary OP cohorts (*p* = 0.09, *p* = 0.37). The absence of heterogeneity in the elderly-onset subgroup (*I*² = 0%) implied that population heterogeneity may underlie variability in outcomes. Acupuncture protocol subgroup analyses identified laser acupuncture as significantly more effective than controls for total BMD improvement (SMD = 0.93, 95% CI = 0.43 to 1.44, *I* ^2^ = NA, *p <*0.001). In contrast, electroacupuncture and warm needle moxibustion demonstrated no significant effects relative to controls (*p* = 0.3, *p* = 0.11) (**Table 2**).

**Table 2:** Outcome indicators of acupuncture and moxibustion for OP.

Subgroup	Change in GA				Change in total BMD				Change in BMD of femoral neck			
	Study	MD [95%CI]	*P* value	*I* ^2^	Study	SMD [95%CI]	*P* value	*I* ^2^	Study	MD [95%CI]	*P* value	*I* ^2^
*Total*	8	0.17 [0.08-0.26]	0.0002	85%	6	0.47 [0.03-0.90]	0.04	86%	8	0.05 [0.01-0.09]	0.01	65%
*Population*												
Postmenopausal	1	0.40 [0.26-0.54]	0.00001	NA	1	0.93 [0.43-1.44]	0.0003	NA	4	0.06 [0.01-0.11]	0.01	62%
Senile	5	0.12 [0.03-0.21]	0.008	81%	3	0.20 [-0.03-0.42]	0.09	0%	1	-0.00 [-0.07-0.06]	0.13	NA
Perimenopausal									1	0.05 [-0.04-0.14]	0.25	NA
Primary	2	0.17 [0.00-0.34]	0.04	55%	2	0.70 [-0.84-2.23]	0.37	96%	2	0.05 [-0.02-0.13]	0.17	36%
*Course of treatment*												
>3 months	2	0.05 [-0.05-0.15]	0.3	69%					3	0.04 [-0.03-0.11]	0.24	87%
≤3 months	6	0.22 [0.11-0.33]	0.0001	79%	5	0.52 [-0.02-1.06]	0.06	88%	5	0.05 [0.01-0.09]	0.02	0%
*Intervention*												
Ordinary acupuncture	2	0.25 [-0.03-0.53]	0.08	91%					3	0.07 [0.01-0.13]	0.02	0%
Electro-acupuncture	3	0.18 [0.03-0.33]	0.02	81%	2	0.76 [-0.67-2.19]	0.3	95%	1	0.02 [-0.06-0.10]	0.62	NA
Warming needle moxibustion	3	0.20 [0.11-0.30]	0.0001	0%	4	0.47 [-0.11-1.05]	0.11	89%	4	0.07 [0.03-0.11]	0.002	58%
Thick needle												
Laser acupuncture					1	0.93 [0.43-1.44]	0.0003	NA				
Acupoint catgut embedding	1	0.01 [-0.04-0.06]	0.69	NA					1	-0.00 [-0.07-0.06]	0.9	NA
Subgroup	Change in BMD of the lumbar spine				Change in BMD of the hip				Change in BGP			
	Study	SMD [95%CI]	*P* value	*I* ^2^	Study	SMD [95%CI]	*P* value	*I* ^2^	Study	SMD [95%CI]	*P* value	*I* ^2^
*Total*	22	0.40 [0.21-0.60]	0.0001	80%	5	0.55 [0.15-0.95]	0.008	83%	9	0.52 [0.07-0.98]	0.02	92%
*Population*												
Postmenopausal	5	0.65 [-0.15-1.45]	0.11	93%					1	-0.61 [-1.04/-0.17]	0.006	NA
Senile	13	0.32 [0.13-0.50]	0.0009	65%	3	0.39 [-0.18-0.96]	0.18	86%	4	0.63 [-0.12-1.38]	0.10	94%
Perimenopausal	1	0.02 [-0.46-0.51]	0.92	NA								
Primary	3	0.57 [0.25-0.90]	0.0005	50%	2	0.82 [0.55-1.09]	0.00001	4%	4	0.69 [0.11-1.26]	0.02	88%
*Course of treatment*												
>3 months	7	0.51 [0.02-1.00]	0.04	91%	1	0.04 [-0.27-0.35]	0.81	NA	3	0.37 [-0.87-1.60]	0.56	97%
≤3 months	14	0.33 [0.16-0.51]	0.0002	57%	3	0.59 [0.14-1.04]	0.0002	71%	6	0.59 [0.22-0.97]	0.002	81%
*Intervention*												
Ordinary acupuncture	10	0.28 [0.09-0.47]	0.004	52%	3	0.25 [-0.08-0.59]	0.14	57%	3	1.06 [0.35-1.77]	0.003	89%
Electro-acupuncture	3	0.63 [0.15-1.11]	0.01	73%					1	1.18 [0.69-1.68]	0.00001	NA
Warming needle moxibustion	10	0.57 [0.18-0.96]	0.004	88%	3	0.87 [0.65-1.09]	0.00001	0%	6	0.41 [-0.12-0.95]	0.19	90%
Thick needle	1	0.08 [-0.36-0.52]										
Laser acupuncture					1	0.93 [0.43-1.44]	0.0003	NA				
Acupoint catgut embedding	1	0.05 [-0.21-0.31]	0.69	NA					1	-0.00 [-0.07-0.06]	0.9	NA

OP, osteoporosis; BMS, MD, mean difference; SMD, standardized mean difference.

#### 3.4.2 Change in BMD of the femoral neck

The improvement of BMD exhibited slight variations amongst the diverse anatomical sites. Eight RCTs evaluated femoral neck BMD changes. Acupuncture outperformed controls with moderate heterogeneity (MD = 0.05, 95% CI = 0.01 to 0.09, *I* ^2^ = 65%, *p* = 0.01) (**Figure 4**). Funnel plot asymmetry (**Figure 5**) and Egger’s test (*p* = 0.026) suggested publication bias, though sensitivity analysis indicated robustness to confounding factors (**Figure 6**).

PMOP patients exhibited greater femoral neck BMD improvements versus controls (MD = 0.06, 95% CI = 0.01 to 0.11, *I* ^2^ = 62%, *p* = 0.01), while elderly-onset, perimenopausal, and POP groups showed no significant differences (*p* = 0.13, *p* = 0.25, *p* = 0.17). Shorter treatment durations (≤ 3 months) were associated with significant femoral neck BMD improvements (MD = 0.05, 95% CI = 0.01 to 0.09, *I* ^2^ = 0%, *p* = 0.02), whereas longer durations (>3 months) showed no effect (*p* = 0.24). Subgroup analysis of acupuncture modalities identified WNM as most effective (MD = 0.07, 95% CI = 0.03 to 0.11, *I* ^2^ = 58%, *p* = 0.002), followed by ordinary acupuncture (MD = 0.07, 95% CI = 0.01 to 0.13, *I* ^2^ = 0%, *p* = 0.02). EA and ACE showed no significant benefits (*p* = 0.62 and *p* = 0.9, respectively) (**Table 2**). Heterogeneity likely stemmed from population characteristics, intervention types, and treatment duration.

#### 3.4.3 Change in BMD of the lumbar

A comprehensive analysis of 22 RCTs confirmed acupuncture’s superiority over controls for lumbar spine BMD improvement (SMD = 0.40, 95% CI= 0.21 to 0.60, *I* ^2^ = 80%, *p* < 0.001) (**Figure 4**). Minor funnel plot asymmetry (**Figure 5**) was noted, but Egger’s test did not indicate statistical bias (*p* = 0.203). Sensitivity analysis confirmed stability across assumptions (**Figure 6**).

Subgroup analyses stratified by diagnosis revealed significant lumbar BMD improvements in subgroups with POP (SMD = 0.32, 95% CI= 0.13 to 0.50, *I* ^2^ = 65%, *p* < 0.001) and SOP (SMD = 0.57, 95% CI= 0.25 to 0.90, *I* ^2^ = 50%, *p* < 0.001). Postmenopausal and perimenopausal groups showed no significant differences (*p* = 0.11, *p* = 0.92). Acupuncture protocol subgroup analyses demonstrated significant lumbar BMD enhancements for ordinary acupuncture, EA, and WNM. EA exhibited the strongest effect (SMD = 0.63, 95% CI= 0.15 to 1.11, *I* ^2^ = 73%, *p* = 0.01), followed by WNM (SMD = 0.57, 95% CI= 0.18 to 0.96, *I* ^2^ = 88%, *p* = 0.004). Treatment duration further influenced outcomes: interventions exceeding 3 months demonstrated greater lumbar BMD improvements (SMD = 0.51, 95% CI= 0.02 to 1.00, *I* ^2^ = 91%, *p* = 0.04) compared to shorter regimens (≤ 3 months: SMD = 0.33, 95% CI= 0.16 to 0.51, *I* ^2^ = 57%, *p* < 0.001) (**Table 2**). No significant sources of heterogeneity were identified in this subgroup.

#### 3.4.4 Change in BMD of the Waed’s triangle

Three RCTs evaluated changes in Ward’s triangle BMD. Acupuncture demonstrated superior efficacy over controls in improving Ward’s triangle BMD (MD = 0.07, 95% CI = 0.01 to 0.13, *I* ^2^ = 72%, *p* = 0.02) (**Figure 4**). Visual inspection of the funnel plot (**Figure 5**) and Egger’s test (*p* = 0.203) indicated no significant publication bias. Sensitivity analysis revealed that exclusion of study (19) attenuated statistical significance to nonsignificant levels (**Figure 6**).

#### 3.4.5 Change in BMD of the hip

Five RCTs assessed hip BMD changes. Acupuncture significantly outperformed controls in improving hip BMD (SMD = 0.55, 95% CI = 0.15 to 0.95, *I* ^2^ = 83%, *p* < 0.001) (**Figure 4**). Funnel plot asymmetry (**Figure 5**) and Egger’s test (*p* = 0.681) showed no evidence of publication bias. Sensitivity analysis identified no major confounding factors (**Figure 6**).

Subgroup analyses stratified by population characteristics revealed significant hip BMD improvements in PMOP patients compared with controls (SMD = 0.82, 95% CI = 0.55 to 1.09, *I* ^2^ = 4%, *p* < 0.001), whereas no differences were observed in the SOP group (*p* = 0.18). Shorter treatment durations (≤ 3 months) showed greater hip BMD improvements with acupuncture versus controls (SMD = 0.59, 95% CI = 0.14 to 1.04, *I* ^2^ = 71%, *p* < 0.001), while longer durations (>3 months) yielded nonsignificant results. Subgroup analyses of acupuncture protocols identified WNM as the most effective intervention for hip BMD improvement (SMD = 0.87, 95% CI = 0.65 to 1.09, *I* ^2^ = 0%, *p* < 0.001) (**Table 2**), suggesting that population characteristics and acupuncture modalities may influence heterogeneity.

### 3.5 Secondary outcomes

#### 3.5.1 Adverse events

**Figure 7:**
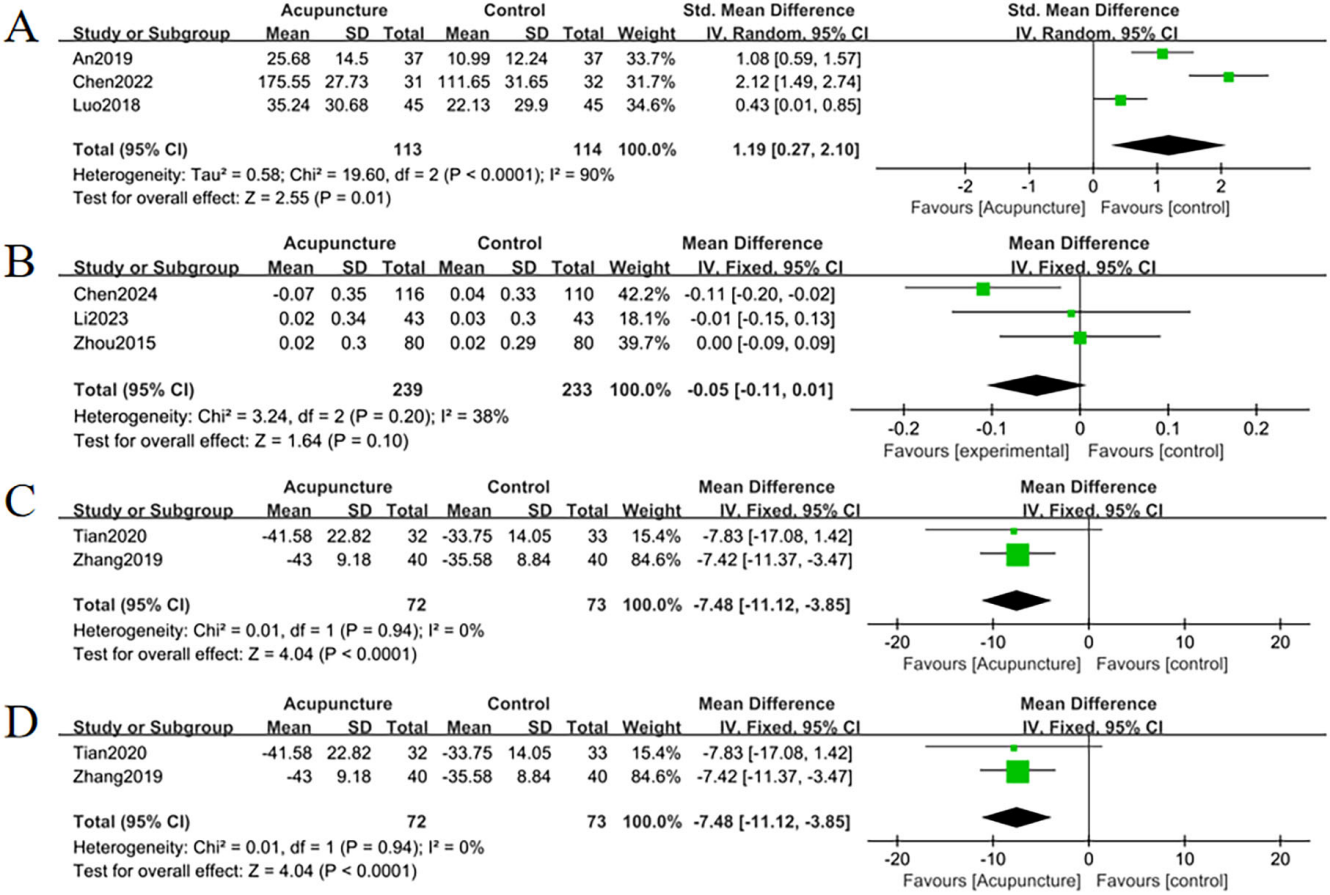
Forest plots of **(A)** change in SF-36 quality of life scale, **(B)** change in P, **(C)** change in DOI, **(D)** adverse events. Forest plots indicated that changes in P levels were not significantly different from controls (*p* = 0.10), while all other outcomes showed significant efficacy (*p <*0.05).

**Figure 8:**
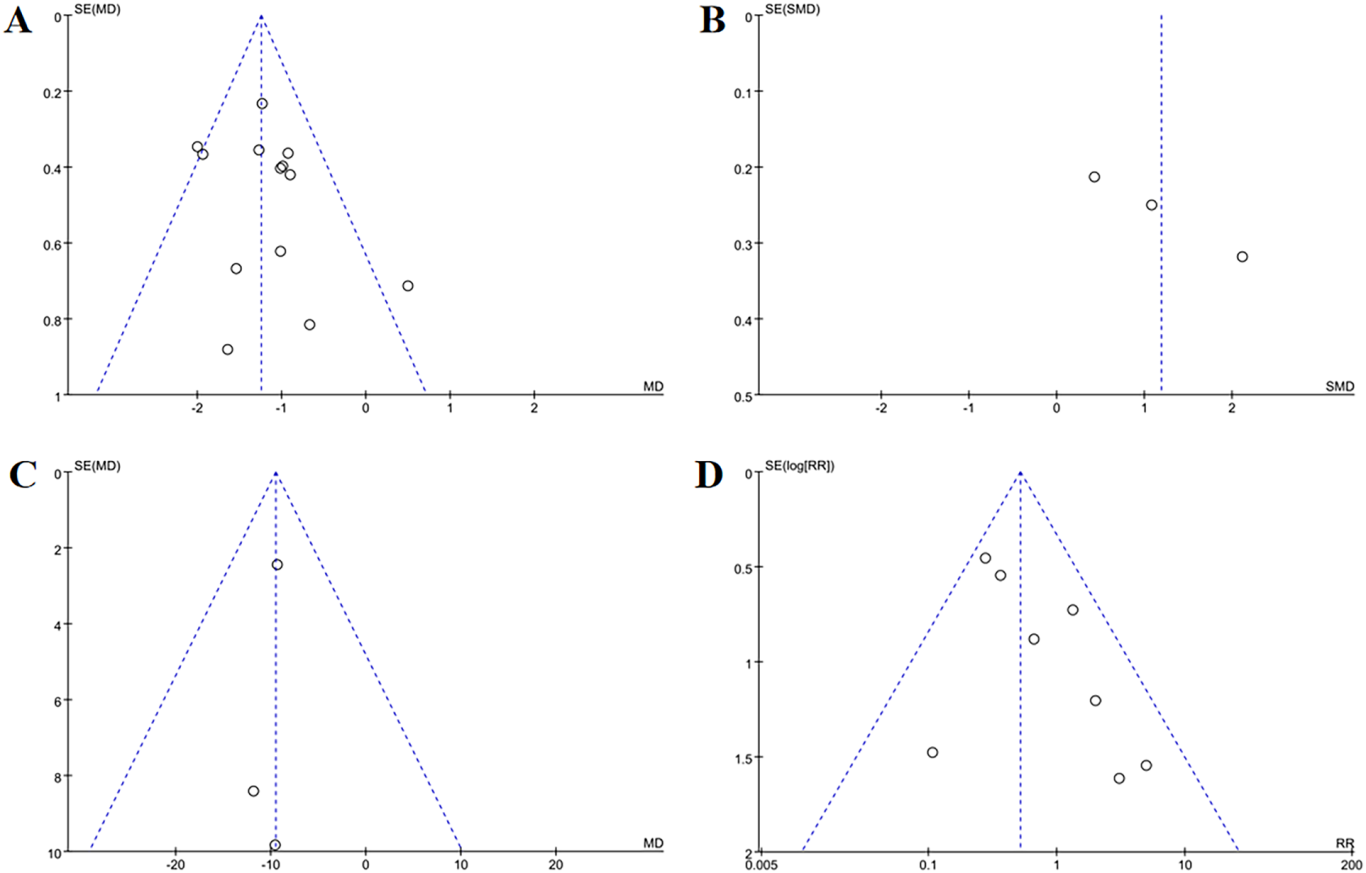
Funnel plots of secondary outcome measures: **(A)** change in VAS **(B)** change in SF-36 quality of life scale, **(C)** change in P, **(D)** adverse events. Funnel plots demonstrated publication bias for SF-36 quality of life scale and adverse events, mild bias for VAS and P levels, and no bias for other outcomes.

Eight studies reported adverse events. Acupuncture significantly reduced adverse event incidence compared to controls (RR = 0.52, 95% CI = 0.32 to 0.84, *I* ^2^ = 29%, *p* = 0.008) (**Figure 7**). Minor funnel plot asymmetry (**Figure 8**) suggested potential publication bias; however, Egger’s test (*p* = 0.100) indicated no statistical significance.

#### 3.5.2 Change in TCM syndrome score

**Figure 9:**
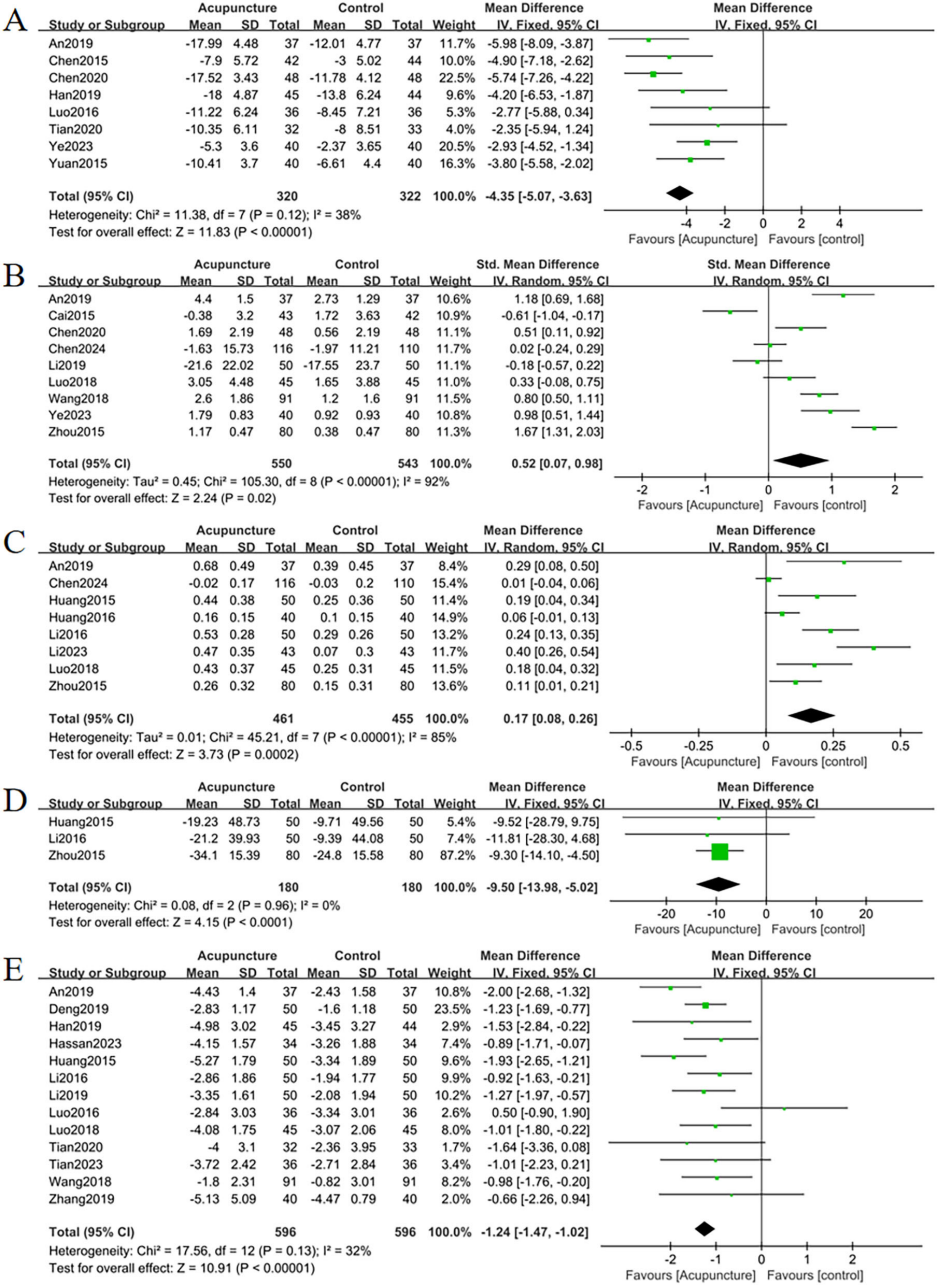
Forest plots of **(A)** change in TCM syndrome score, **(B)** change in GA, **(C)** change in BGP, **(D)** change in ALP, **(E)** change in VAS. Forest plots demonstrated that all outcomes were significantly more effective than controls (*p <*0.05).

**Figure 10:**
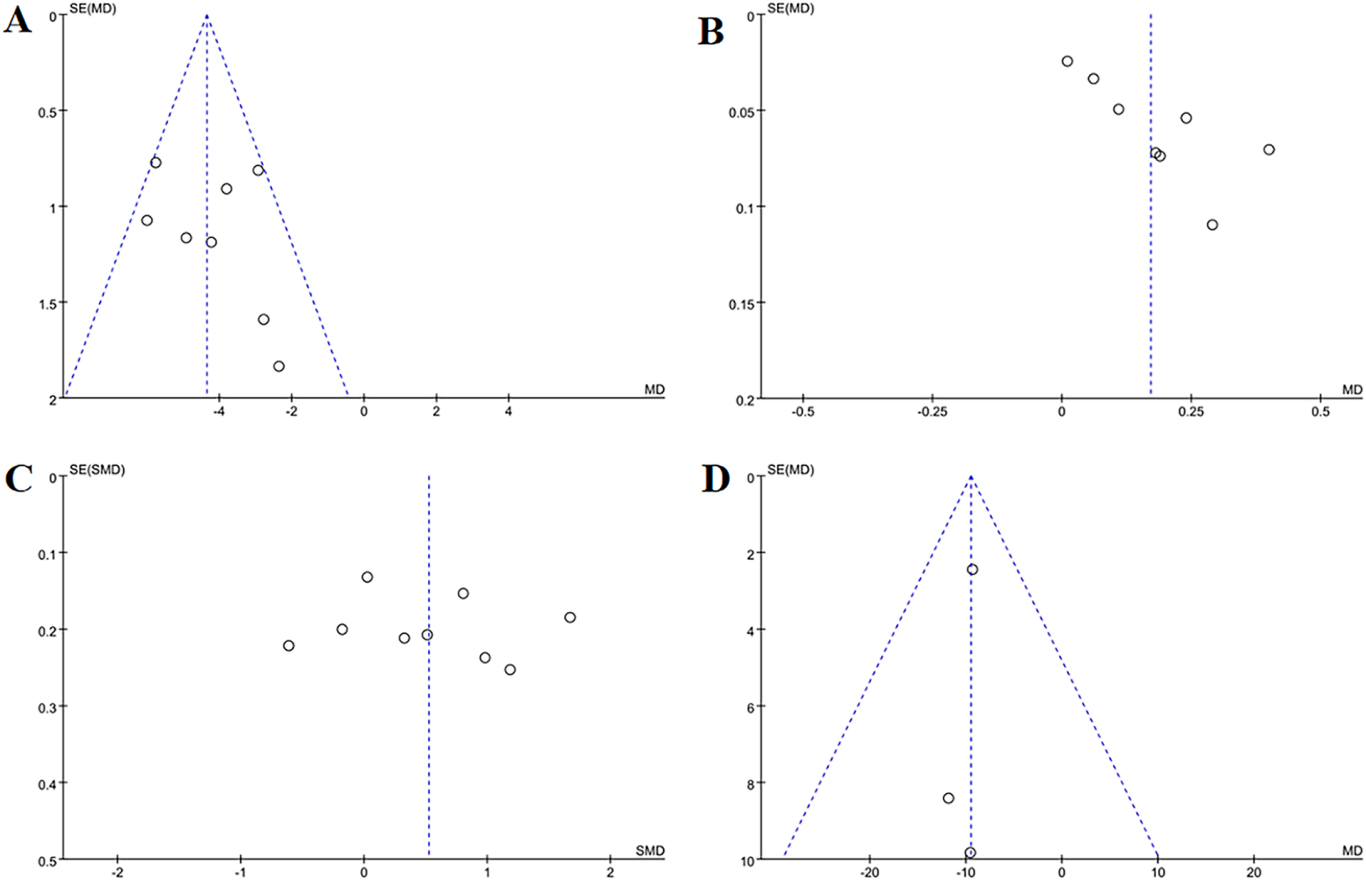
Funnel plots of secondary outcome measures: **(A)** change in TCM syndrome score, **(B)** change in GA, **(C)** change in BGP, **(D)** change in ALP. Funnel plots revealed publication bias for changes in GA and TCM syndrome scores, mild bias for BGP and ALP, and no bias for P levels.

Eight studies analyzed changes in TCM syndrome scores. Acupuncture demonstrated superior efficacy over controls in alleviating TCM syndrome scores (MD = -4.35, 95% CI = -5.07 to -3.63, *I* ^2^ = 38%, *p* < 0.001) (**Figure 9**). Minimal funnel plot asymmetry (**Figure 10**) and Egger’s test (*p* = 0.458) confirmed the absence of publication bias.

#### 3.5.3 Change in GA

**Figure 11:**
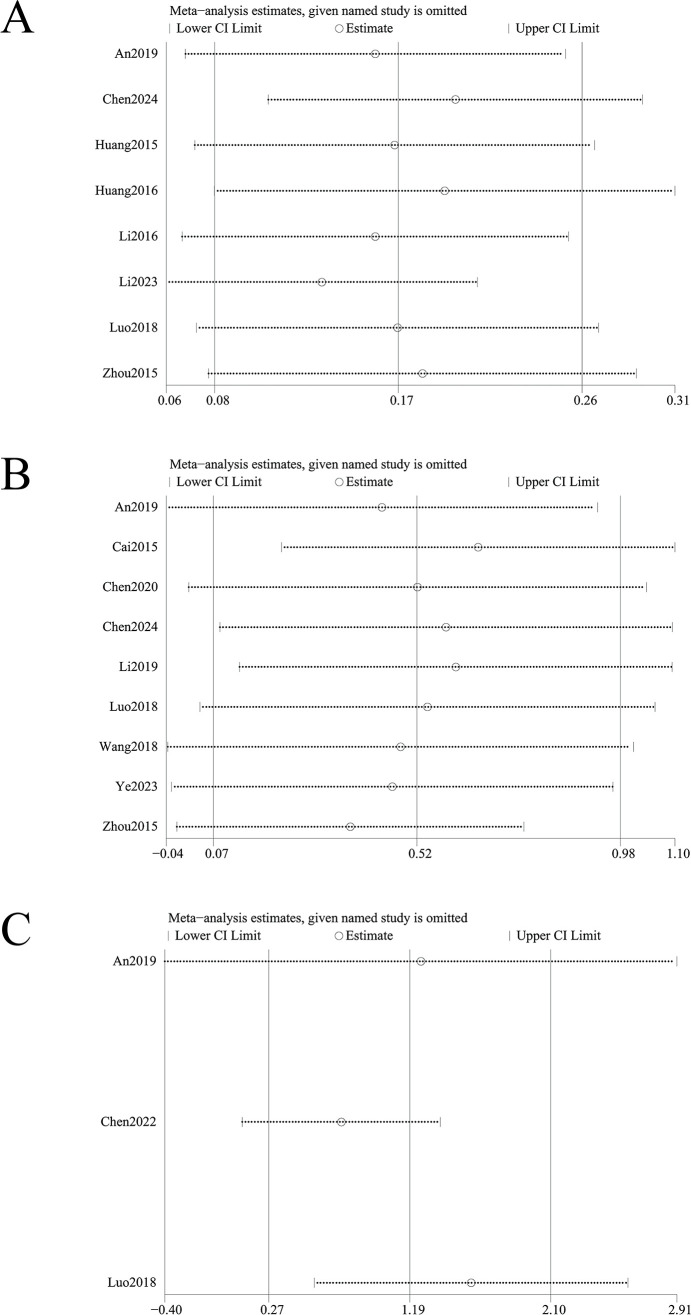
Sensitivity analysis of **(A)** change in GA, **(B)** change in BGP, **(C)** change in SF-36 quality of life scale. Sensitivity analysis identified instability in outcomes of changes in BGP and SF-36 quality of life scale, whereas changes in GA showed stability.

Eight studies evaluated changes in GA levels. Acupuncture demonstrated superior efficacy over controls in improving GA levels (MD = 0.17, 95% CI = 0.08 to 0.26, *I* ^2^ = 85%, *p* < 0.001) (**Figure 9**). Funnel plot asymmetry (**Figure 10**) and Egger’s test (*p* = 0.005) suggested potential publication bias; however, sensitivity analysis confirmed robustness to confounding factors (**Figure 11**).

Shorter treatment durations (≤ 3 months) showed greater GA level reductions with acupuncture versus controls (MD = 0.22, 95% CI = 0.11 to 0.33, *I* ^2^ = 79%, *p* < 0.001), whereas longer durations (>3 months) yielded nonsignificant results. Subgroup analyses of acupuncture protocols identified EA and WNM as most effective. WNM demonstrated the strongest effect (MD = 0.20, 95% CI = 0.11 to 0.30, *I* ^2^ = 0%, *p* < 0.001), followed by EA (MD = 0.18, 95% CI = 0.03 to 0.33, *I* ^2^ = 81%, *p* = 0.02). Conventional acupuncture and ACE showed no significant differences versus controls (*p* = 0.08 and *p* = 0.69, respectively). These findings suggest that acupuncture protocol variability may influence heterogeneity. Acupuncture significantly improved GA levels across diverse OP populations, with the POP subgroup exhibiting the most pronounced reduction (MD = 0.40, 95% CI = 0.26 to 0.54, *I* ^2^ = NA, *p* < 0.001) (**Table 2**).

#### 3.5.4 Change in BGP

Nine studies assessed changes in BGP levels. Acupuncture significantly outperformed controls in improving BGP levels (SMD = 0.52, 95% CI = 0.07 to 0.98, *I* ^2^ = 92%, *p* = 0.02) (**Figure 9**). Funnel plot asymmetry (**Figure 10**) was minimal, and Egger’s test confirmed no publication bias (*p* = 0.697). Sensitivity analysis revealed that exclusion of studies 24,31,32 and 43 attenuated statistical significance to nonsignificance (**Figure 11**).

Population-based subgroup analyses demonstrated significant BGP improvements in POP patients versus controls (SMD = 0.69, 95% CI = 0.11 to 1.26, *I* ^2^ = 88%, *p* = 0.02), while no differences were observed in SOP patients (*p* = 0.10). Shorter treatment durations (≤ 3 months) showed greater BGP enhancements with acupuncture versus controls (SMD = 0.59, 95% CI = 0.22 to 0.97, *I* ^2^ = 81%, *p* = 0.002), whereas longer durations (>3 months) had no significant effect (*p* = 0.56).

Subgroup analyses of acupuncture protocols identified EA and conventional acupuncture as most effective. EA exhibited the strongest effect (SMD = 1.18, 95% CI = 0.69 to 1.68, *I* ^2^ = NA, *p* < 0.001), followed by simple acupuncture (SMD = 1.06, 95% CI = 0.35 to 1.77, *I* ^2^ = 89%, *p* = 0.003). WNM and ACE showed no significant benefits (*p* = 0.19 and *p* = 0.85, respectively) (**Table 2**). Subgroup analyses did not identify significant sources of heterogeneity.

#### 3.5.5 Change in ALP

Three studies evaluated changes in ALP levels. Acupuncture demonstrated a significant reduction in ALP levels compared to controls (MD = -9.05, 95% CI= -13.98 to -5.02, *I* ^2^ = 0%, *p* < 0.001) (**Figure 9**). There were no signs suggesting publication bias exists based on the funnel plot (**Figure 10**) as well as Egger’s test (*p* = 0.496).

#### 3.5.6 Change in VAS

Thirteen studies assessed pain relief using VAS scores. Acupuncture exhibited superior pain reduction compared to controls (MD = -1.24, 95% CI = -1.47 to -1.02, *I* ^2^ = 32%, *p* < 0.001) (**Figure 9**). Minimal funnel plot asymmetry (**Figure 10**) and Egger’s test (*p* = 0.322) confirmed the absence of publication bias.

#### 3.5.7 Change in SF-36 quality of life scale

Three studies analyzed improvements in SF-36 quality of life scores. Acupuncture significantly enhanced quality of life versus controls (SMD = 1.19, 95% CI = 0.27 to 2.10, *I^2^* = 90%, *p* < 0.01) (**Figure 7**). While funnel plot asymmetry (**Figure 8**) and Egger’s test (*p* = 0.032) suggested potential publication bias, sensitivity analysis revealed that exclusion of study (32) attenuated statistical significance to nonsignificance (**Figure 11**).

#### 3.5.8 Change in P

Three studies evaluated serum phosphorus levels. No significant differences were observed between acupuncture and controls (MD=-0.05, 95% CI= -0.11 to 0.01, *I^2^ =* 38%, *p*=0.10) (**Figure 7**). There were no signs suggesting publication bias exists based on the funnel plot (**Figure 8**) as well as Egger’s test (*p* = 0.734).

#### 3.5.9 Change in ODI

The outcome index analysis of ODI score alterations comprised two studies. The findings indicated that the efficacy of acupuncture modalities significantly surpassed the efficacy of the control group in declining the score of the DOI (MD = -7.48, 95% CI = -11.12 to -3.85, *I^2^* = 0%, *p* < 0.001) (**Figure 7**).

#### 3.5.10 Efficacy evaluation of TCM syndromes

**Figure 12:**
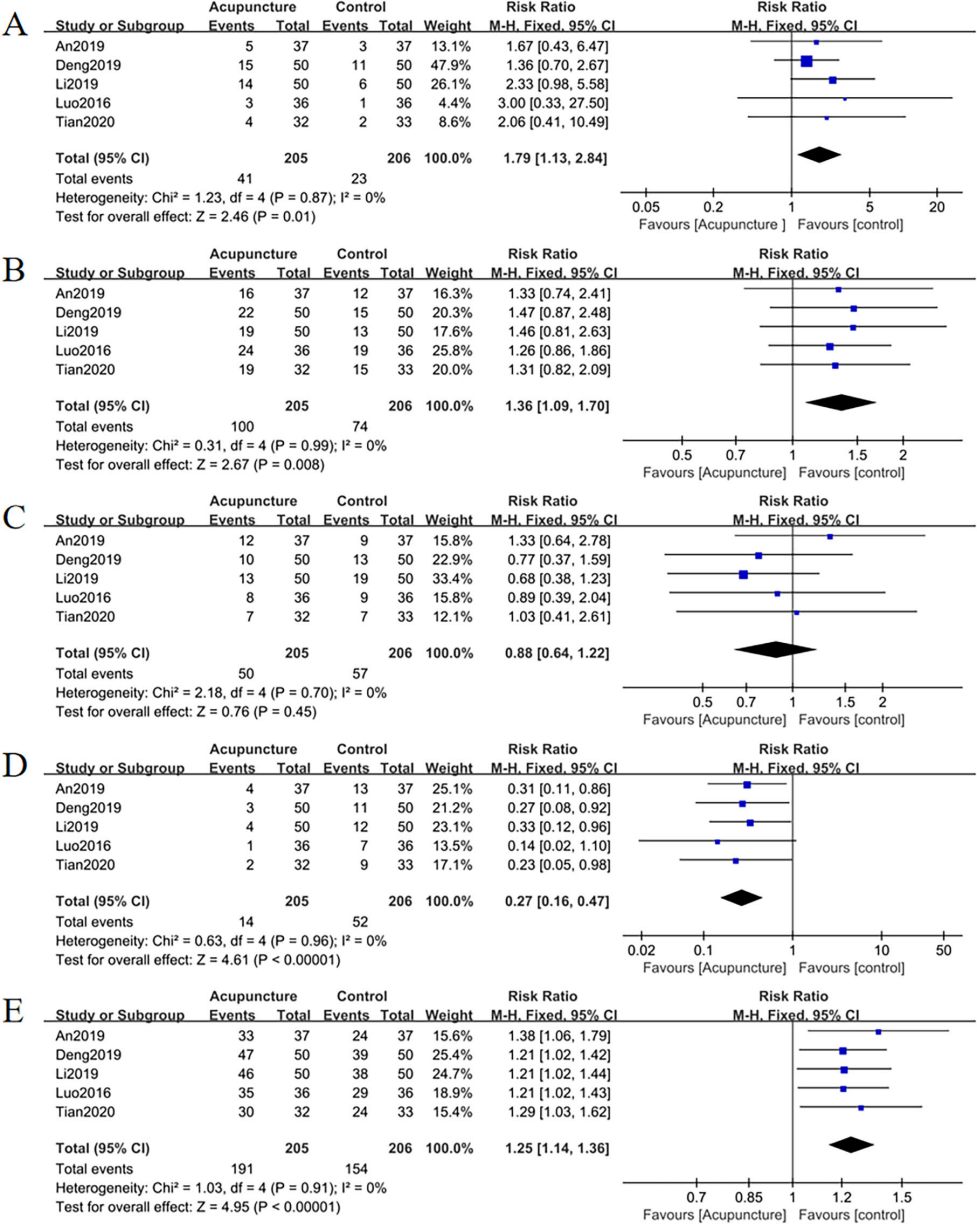
Forest plots of TCM syndrome efficacy evaluation **(A)** total effective rate, **(B)** cured, **(C)** markedly improved, **(D)** improved, **(E)** failure. Forest plots revealed significant increases in total effective rate, cured, and markedly improved cases compared to controls (*p <*0.05). Improved cases showed no significant difference (*p* = 0.45), while failure cases were significantly reduced (*p* <0.05).

Five studies analyzed TCM syndrome outcomes. Acupuncture demonstrated superior efficacy across multiple metrics:

​Total effective cases: RR = 1.25, 95% CI = 1.14 to 1.36, *I^2^* = 0%, *p* = 0.01;

Cured cases: RR = 1.79, 95% CI = 1.13 to 2.84, *I^2^* = 0%, *p* = 0.01;

Markedly improved cases: RR = 1.36, 95% CI = 1.09 to 1.70, *I^2^* = 0%, *p* = 0.008;

Improved cases: RR = 0.88, 95% CI = 0.64 to 1.22, *I^2^* = 0%, *p* = 0.45;

Failure cases: RR = 0.27, 95% CI = 0.16 to 0.47, *I^2^* = 0%, *p* < 0.001 (**Figure 12**).

**Figure 13:**
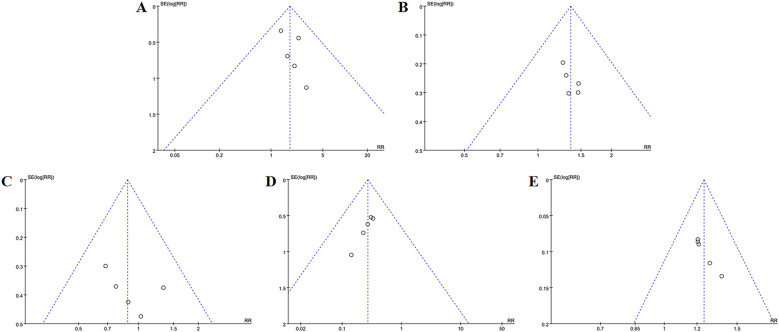
Funnel plots of TCM syndrome efficacy evaluation **(A)** total effective rate, **(B)** cured, **(C)** markedly improved, **(D)** improved, **(E)** failure. In TCM syndrome efficacy evaluation, funnel plots indicated publication bias for total effective rate, cured, and failure categories, but no bias for markedly improved or improved outcomes.

​Funnel plots (**Figure 13**) as well as Egger’s test indicated publication bias for total effective cases (*p* = 0.007), cured cases (*p* = 0.040) and failure cases (*p* = 0.010), but no bias for markedly improved cases (*p* = 0.040) and improved case numbers (*p* = 0.323).

#### 3.5.11 Efficacy evaluation of osteoporosis

**Figure 14:**
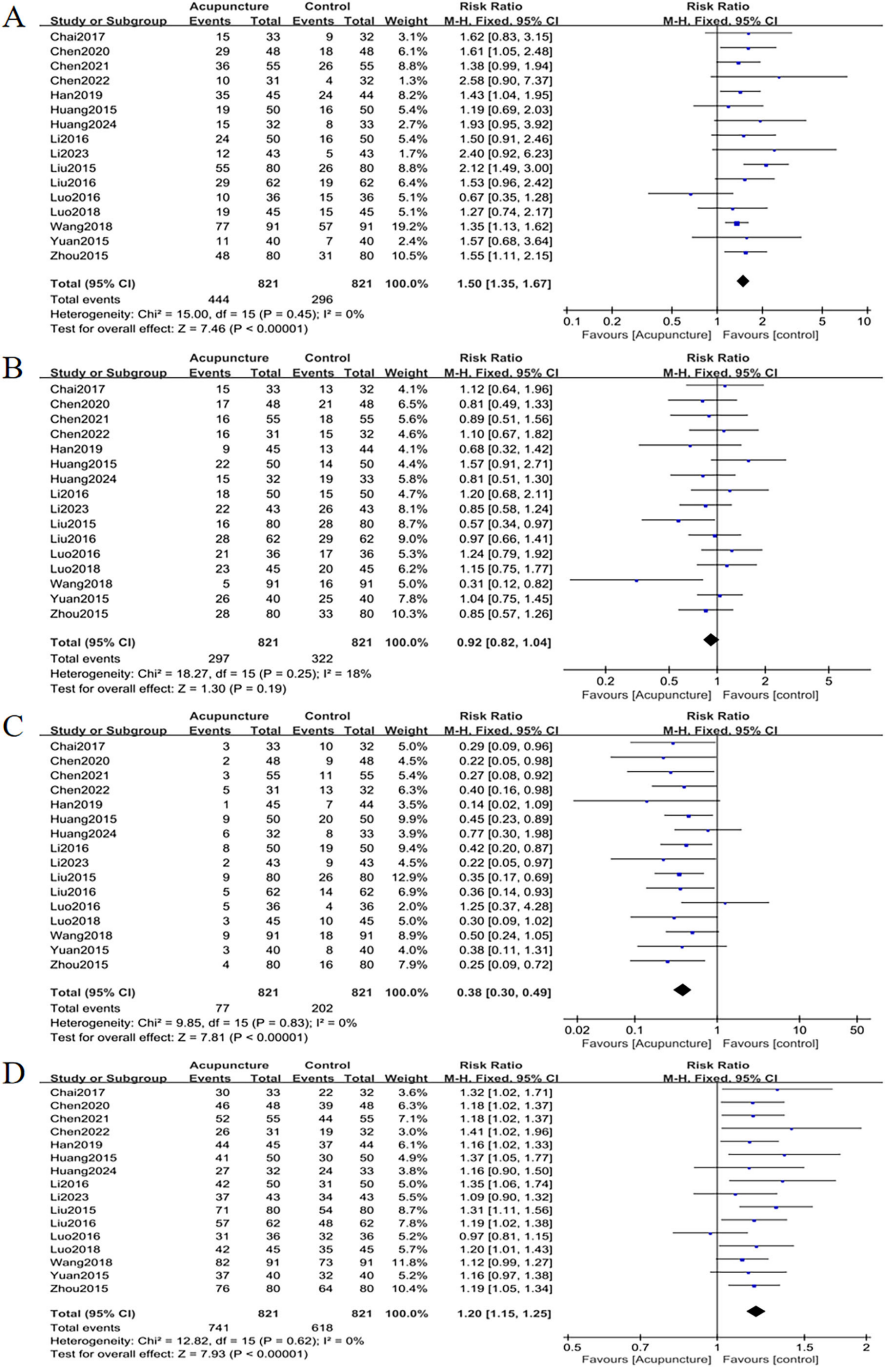
Forest plots of efficacy evaluation of osteoporosis **(A)** total effective rate, **(B)** cured, **(C)** improved, **(D)** failure. Forest plots demonstrated significant increases in total effective rate and cured cases versus controls (p <0.05). Improved cases had no significant difference (p = 0.19), while failure cases were markedly lower (*p* <0.05).

Sixteen studies evaluated OP treatment outcomes. Acupuncture significantly outperformed controls:

Total effective cases: RR = 1.20, 95% CI = 1.15 to 1.25, *I^2^* = 0%, *p* < 0.001;

Markedly improved cases: RR = 1.50, 95% CI = 1.35 to 1.67, *I^2^* = 0%, *p* < 0.001;

Improved cases: RR = 0.92, 95% CI = 0.82 to 1.04, *I^2^* = 18%, *p* = 0.19;

Failure cases: RR = 0.38, 95% CI = 0.30 to 0.49, *I* ^2^ = 0%, *p* < 0.001 (**Figure 14**).

**Figure 15:**
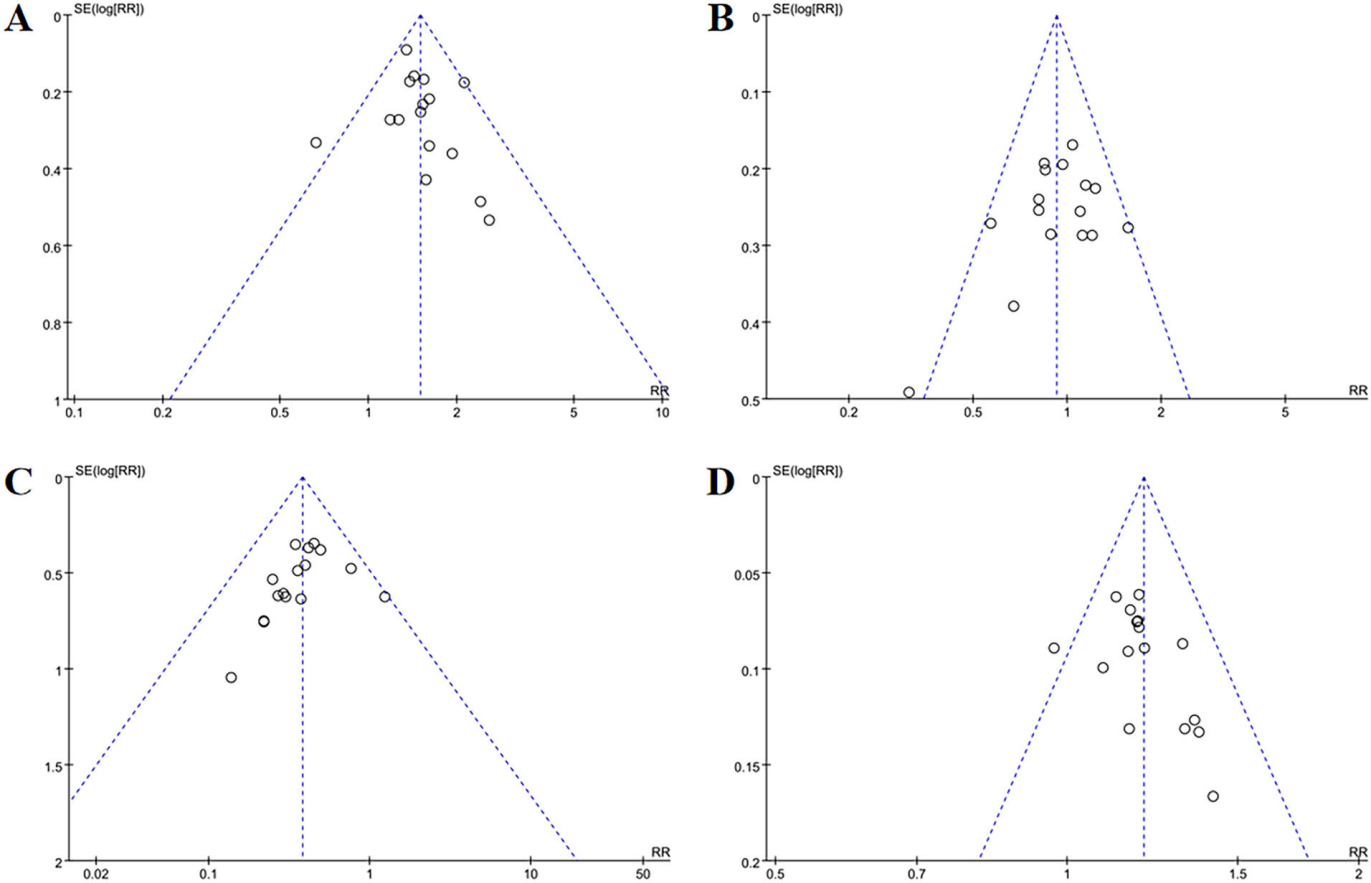
Funnel plots of efficacy evaluation of osteoporosis **(A)** total effective rate, **(B)** cured, **(C)** improved, **(D)** failure. For osteoporosis treatment efficacy, funnel plots showed mild publication bias for total effective rate, cured, and failure, while improved outcomes had no bias.

Funnel plots (**Figure 15**) showed minor publication bias for total effective cases, markedly improved cases and failure case, though Egger’s test confirmed no statistical significance (*p* = 0.088, *p* = 0.452, *p* = 0.145). There were no signs suggesting publication bias in improved cases (*p* = 0.168).

## 4 Discussion

Previous reviews (17) have indicated that acupuncture, both as a therapeutic and adjunctive treatment, has proven particularly effective in maintaining bone mass and alleviating clinical symptoms in OP patients. However, while there is some evidence to suggest the use of acupuncture as a treatment for OP, it is not entirely conclusive. The number of clinical trials is limited and the methodological quality is not optimal. The objective of this study is to carry out such a comprehensive evaluation, gaining a more in-depth understanding of how acupuncture performs in terms of both improving the condition of OP patients and ensuring their safety during the treatment process. To this end, we will be incorporating recent RCTs from an extensive search.

As outlined in the review, an analysis was conducted on 28 studies, encompassing collectively 2,758 patients. In comparison to the 2018 meta-analysis by Pan et al., the literature in our review is deemed more innovative and of superior quality. Apart from POP and PMOP, our study also incorporated SOP. The age range of the participants is comparable, with a typical range of approximately 50 to 70 years. Two studies suggested that acupuncture is beneficial for OP patients, reporting improvements in BMD, GA, ALP, clinical efficacy rate, and pain relief. Furthermore, our research included additional outcome measures such as BGP, ODI, SF-36 scores, TCM syndrome scores, and adverse event reports. The results imply that acupuncture improves these indicators in OP and reduces the risk of adverse events when integrated with traditional medications. The primary acupuncture points examined are along the BL, GB, and GV meridians, with BL23, BL20, and ST36 being the most frequently used points, in line with Pan et al.’s analysis. Additionally, the use of GB39 in treating OP has seen an increase in recent years. TCM considers GB39 as part of the GB meridian, known for its role in strengthening bones and alleviating pain (47). Recent studies (48) indicate that acupuncture points on the GB meridian, including GB39, may affect bone mass via sympathetic and sensory nerve pathways, contributing to maintaining the balance between bone formation and resorption, thus positioning GB39 as a promising acupuncture point for OP treatment

Heterogeneity analysis revealed that outcome measures, including OP efficacy evaluations, TCM syndrome efficacy assessments, changes in ODI, pain scale alterations, ALP, and TCM syndrome score variations, demonstrated low heterogeneity, implying reliable results. Conversely, higher heterogeneity was noted in changes in total BMD, changes in Ward’s triangle BMD, femoral neck BMD, lumbar BMD, hip BMD, GA, BGP, and SF-36 scores. Subsequent subgroup analyses indicate that these variations may be related to differences in acupuncture techniques, combined treatment methods, intervention durations, and control measures. Future studies should strive to standardize research designs to address these factors and reduce heterogeneity.

Sensitivity analysis indicated that excluding specific studies altered the statistical differences for changes in total BMD, BGP, Ward’s triangle BMD, and SF-36 outcome measure. This suggests that these studies significantly influenced the overall results and contributed to high heterogeneity. The high heterogeneity observed in other outcome measures persisted after the sensitivity analysis, suggesting that the contributing factors may be numerous and complex. Therefore, it is crucial to objectively assess the effects of acupuncture on these outcome measures with high heterogeneity. For optimal efficiency, recovery, and effectiveness in changes in BMD, femoral neck BMD, GA, SF-36 scoring, and TCM syndrome efficacy evaluation, publication bias was present, likely due to the homogeneity of patient demographics, small sample sizes, and variations in treatment duration. This publication bias may result in overestimating existing research findings, thereby impacting the reliability of the results. Following comprehensive analysis, no evidence of significant publication bias was identified in the other results.

The substantial heterogeneity observed across key outcomes (*I* ^2^ = 65-85% for BMD metrics) and identified publication bias present significant challenges to interpreting these findings. Notably, the preponderance of small-scale trials from China (79% of included studies with n < 100) may have inflated effect estimates, thereby compromising the validity of pooled effect estimates. This methodological variability, compounded by potential regional selection bias, underscores the necessity for cautious extrapolation of findings to broader, more diverse populations.

Detailed subgroup analyses identified potential sources of high heterogeneity in particular contexts. In order to investigate the various factors which may contribute to treatment effect heterogeneity, this study explores the effects of different acupuncture interventions, specific OP populations and various intervention durations. We found that differences in the intervention population could account for the high heterogeneity in outcome measures, including changes in total BMD, femoral neck BMD, and hip BMD. Variations in acupuncture techniques contributed to the high heterogeneity observed in outcome measures, including changes in GA and femoral neck BMD. Additionally, treatment duration significantly impacted changes in femoral neck BMD.

The robustness of evidence indicates that acupuncture demonstrates superior efficacy compared to conventional therapies in OP patients during short-term interventions (≤3 months), with no significant benefits observed for prolonged regimens (>3 months) in improving BMD, GA, or BGP. This pattern suggests that acupuncture primarily exerts rapid, transient effects on osteogenic pathways, potentially mediated by immediate activation of bone remodeling mechanisms (49, 50). Prolonged treatment protocols may reduce adherence and elevate adverse event risks (51), supporting the rationale for a time-limited therapeutic window. Clinically, short-term intensive acupuncture (≤3 months) could serve as an adjunctive therapy to rapidly stimulate osteogenesis and mitigate bone loss in early-stage OP or high-fracture-risk populations. For sustained benefits, graduated protocols–such as transitioning to biweekly or monthly maintenance sessions–should be combined with lifestyle modifications (e.g., calcium supplementation, resistance training) to enhance adherence and preserve outcomes.

Our findings reveal distinct therapeutic effects across acupuncture modalities in OP. WNM demonstrated superior efficacy in improving BMD compared to other techniques, while EA exhibited enhanced capacity to elevate GA and BGP levels. WNM integrates thermal stimulation with acupuncture, synergistically amplifying osteogenic effects through localized heat-induced vasodilation and metabolic activation. This aligns with meta-analytic evidence (52) and the findings of Pan et al.,supporting WNM as a first-line intervention for PMOP, particularly in patients with low baseline BMD or comorbid chronic pain.

EA, which combines needle insertion with pulsatile electrical stimulation, modulates neural and systemic pathways to enhance bone remodeling. Preclinical studies (53) demonstrated that EA ameliorates OP in ovariectomized rats by regulating neuroendocrine-immune networks and gut microbiota composition, thereby improving osteoblast activity. However, while EA rapidly improves biochemical markers such as GA/BGP, its effects on BMD manifest more gradually (54). Clinical variability in EA protocols–spanning acupoint selection, stimulation frequency, and treatment duration–necessitates standardized parameters to optimize therapeutic consistency and reproducibility.

Emerging modalities, including thick-needle therapy and laser acupuncture, showed limited efficacy in this review, underscoring the need for further validation through rigorous clinical trials. Despite its potential, laser acupuncture remains underexplored due to insufficient high-quality evidence and variability in technical parameters. These findings emphasize the importance of tailoring acupuncture approaches to patient-specific needs, osteoporosis subtype, and biomarker profiles, while prioritizing standardized protocols for emerging techniques to establish robust clinical guidelines.

This study identified distinct acupuncture efficacy patterns across OP subtypes through subgroup analyses, aligning with Pan’s research and demonstrating substantial differential effects. PMOP, the most prevalent form of OP linked to estrogen deficiency post-menopause (55), arises from hormonal shifts disrupting the balance between bone resorption by OCs and bone formation by OBs. This imbalance results in net bone loss due to excessive osteoclastic activity relative to osteoblastic bone formation (56). Atsushi et al. (57) demonstrated that EA at the Shenshu (BL23) acupoint in OVX rats significantly elevated serum estrogen (E2) levels and BMD after 17 weeks, accompanied by increased testosterone in sham–operated controls. Estrogen’s bone-protective effects are attributed to its direct regulation of OB differentiation, osteocyte viability, and OC activity (58).

Our findings highlight acupuncture’s potential in managing PMOP by improving BMD, particularly in patients with low baseline bone density or comorbidities (e.g., chronic pain). In contrast, SOP, driven by age-related increases in OC activity (59) and accelerated senescence of OBs, BMSCs, and osteocytes (60), leads to disrupted bone remodeling. Zhong et al. (62) demonstrated that EA inhibits cellular senescence in OBs and chondrocytes via the p53/p21 signaling pathway, thereby enhancing BMD. For SOP, combination therapies (e.g., acupuncture plus bisphosphonates or resistance training) may address multifactorial pathogenesis by targeting underlying aging mechanisms (61). These findings underscore the importance of subtype-specific acupuncture strategies to optimize therapeutic outcomes.

Subgroup analysis results provide critical insights for clinicians to optimize acupuncture protocols to individual patient profiles. Adopting a precision medicine approach, practitioners should customize therapeutic modalities, treatment durations, and outcome measures based on OP subtype, biomarker signatures, and patient adherence capacity. Leveraging these subgroup findings, future research should prioritize conducting large-scale RCTs to validate therapeutic efficacy while investigating novel treatment protocols–such as refining EA stimulation parameters or combining multimodal interventions–to enhance clinical applicability and translational relevance.

In summary, acupuncture, when employed as an adjuvant therapy for OP, demonstrates significant efficacy in improving BMD, GA levels, optimizing bone metabolism, pain alleviation, and symptom management compared to conventional treatments. However, several critical limitations must be addressed to contextualize these findings. First, 97.53% of participants were recruited from China, raising concerns about generalizability to diverse populations. Second, regional variations in calcium/vitamin D supplementation regimens were underreported, complicating isolated assessments of acupuncture’s biological effects. Third, overrepresentation of small-scale trials and lack of standardized acupuncture protocols may have contributed to observed heterogeneity. Regional selection bias, small sample sizes, non-standardized interventions, and inherent instability of pooled estimates underscore the need for high-quality, multicenter, rigorously designed RCTs to validate these findings.

## Data Availability

The original contributions presented in the study are included in the article/supplementary material, further inquiries can be directed to the corresponding authors.
